# Retraction Note: FoxM1-mediated RFC5 expression promotes temozolomide resistance

**DOI:** 10.1007/s10565-024-09887-0

**Published:** 2024-06-08

**Authors:** Wan-xin Peng, Xiu Han, Chun-li Zhang, Lu Ge, Feng-yi Du, Jie Jin, Ai-hua Gong

**Affiliations:** 1 School of Medicine, Jiangsu University, Zhenjiang, 212013 Jiangsu China; 2 Department of Gastroenterology, Affiliated Hospital of Jiangsu University, Zhenjiang, 212001 Jiangsu China

**Retraction Note: Cell Biol Toxicol (2017) 33:527–537**

10.1007/s10565-017-9381-1

The Editor-in-Chief has retracted this article after concerns were raised about some of the data reported. Specifically, there is evidence of a potential splice in each of the FoxM1 and RFC5 blots presented in Fig. 6C. The Editor-in-Chief therefore no longer has confidence in the reliability of the data presented.

Ai-hua Gong agrees with this retraction. The remaining authors did not respond to correspondence from the Publisher about this retraction.

